# Exercise Training in Children and Adolescents with Cystic Fibrosis: Theory into Practice

**DOI:** 10.1155/2010/670640

**Published:** 2010-09-19

**Authors:** Craig A. Williams, Christian Benden, Daniel Stevens, Thomas Radtke

**Affiliations:** ^1^Children's Health and Exercise Research Centre, School of Sport and Health Sciences, University of Exeter, Exeter EX1 2LU, UK; ^2^Division of Pulmonary Medicine, University Hospital Zurich, 8091 Zurich, Switzerland

## Abstract

Physical activity and exercise training play an important role in the clinical management of patients with cystic fibrosis (CF). Exercise training is more common and recognized as an essential part of rehabilitation programmes and overall CF care. Regular exercise training is associated with improved aerobic and anaerobic capacity, higher pulmonary function, and enhanced airway mucus clearance. Furthermore, patients with higher aerobic fitness have an improved survival. Aerobic and anaerobic training may have different effects, while the combination of both have been reported to be beneficial in CF. However, exercise training remains underutilised and not always incorporated into routine CF management. We provide an update on aerobic and anaerobic responses to exercise and general training recommendations in children and adolescents with CF. We propose that an active lifestyle and exercise training are an efficacious part of regular CF patient management.

## 1. Introduction

Physical activity and exercise training have become increasingly important and widely accepted as part of therapy and rehabilitation programmes in cystic fibrosis (CF) management. Physical activity refers to any body movement produced by the skeletal muscles and occurs in a variety of forms (i.e., free play, exercise, organised sports), resulting in a substantial increase in energy expenditure [1]. Exercise training, however, can be defined as regular participation in vigorous physical activity to improve physical performance or cardiovascular function or muscle strength or any combination of these three [2]. Prior to initiation of any exercise training, detailed exercise testing is recommended, not only to monitor disease progression, but also to detect exercise-induced limitations and therefore to provide the patients with safe training recommendations [3, 4]. Ideally, exercise training should complement current therapies in CF patient healthcare. In previous reports, however, it has emerged that clinicians lack specific recommendations to instruct their patients appropriately [5, 6]. 

Several studies report beneficial effects of exercise training on cardiopulmonary fitness (CPF) in patients with CF [7–11]. CPF pertains to the functions of both the heart and the pulmonary system, usually expressed as peak oxygen consumption (peak $\dot{\text{V}}{\text{O}}_{2}$), the most reliable and reproducible measure of CPF following maximal exercise testing [12]. Recently, Bradley and Moran examined the effectiveness of exercise training in CF using a systematic Cochrane review [13]. Seven randomised controlled trials with a total number of 231 participants were included, investigating the effects of different types of training (aerobic versus anaerobic training, anaerobic training, aerobic training, and the combination of both). The authors conclude that exercise training is an important part of CF care; however, there are insufficient numbers of studies published to date to document the benefits of exercise training for people with CF. Nevertheless, the authors state that there is no evidence to actively discourage exercise training [13]. In 2009 Wilkes et al. underlined the importance of exercise and habitual physical activity in children with CF [14]. In particular, Wilkes et al. highlighted that education of health care providers and implementing exercise in clinical practice are needed [14]. Regular exercise training may improve pulmonary function, increase aerobic and anaerobic capacity, strengthen ventilatory muscles, and help airway sputum clearance, the latter probably due to enhanced ventilation and vibrations during exercise, leading to mechanical airway clearance [7, 8, 11, 15–19]. Furthermore, it is suspected that moderate intensity exercise blocks the amiloride-sensitive sodium channels of the respiratory epithelium, thus resulting in lower mucus viscosity, simplifying mucus expectoration [20]. Moreover, higher activity levels and peak $\dot{\text{V}}{\text{O}}_{2}$ are positively related to survival in CF [21–23]. Types of training (aerobic versus anaerobic) may have different effects on improvement in exercise tolerance [13], but most training studies so far have focused on endurance training, observing improvements in pulmonary function, peak $\dot{\text{V}}{\text{O}}_{2}$, breathlessness, and quality of life [11, 18, 19, 24]. Exercise training including anaerobic (strength) exercises can improve muscle strength and muscle size, resulting in weight gain [16, 19, 25]. Therefore, a comprehensive training programme should include a variety of sports activities, adapted to the special needs and preferences of each individual. However, the effects of training most likely depend on the training methods, as improvements may only persist with continuous training in the long term. The aim of this paper is to provide a focused update on aerobic and anaerobic responses to exercise and habitual physical activity exclusive to children and adolescents with CF. This paper will help to strengthen the rationale for the initiation of further clinical trials examining the effects of regular exercise on overall health in the paediatric CF population. 

## 2. Method of Review

All relevant studies for this paper were identified using electronic search of Medline and PubMed databases. A bibliography search of all accessed publications was also performed. Key descriptors were cystic fibrosis, training, rehabilitation, aerobic fitness, children, and adolescents. The selection of studies presented in the paper was based upon the agreement by all four authors. We include studies of subjects with CF, independently of their disease severity, and type of exercise training (aerobic training; anaerobic training; combination of aerobic and anaerobic training, as well as inspiratory muscle training). 

### 2.1. Physiological Responses to Exercise in Children and Adolescents with Cystic Fibrosis

Physical activity is a fundamental part of the growth and development of children. Neither any physical activity nor an exercise task can be considered as solely aerobic or anaerobic. The nature of a child's activity requires an interplay of both aerobic and anaerobic metabolism. An update on aerobic and anaerobic performance in children with CF is outlined below. 

#### 2.1.1. Aerobic Responses to Exercise

Aerobic exercises include any kind of activities using large muscle groups that can be maintained continuously in a rhythmic manner (e.g., swimming, cycling, or running). Activities with moderate effort can be performed over hours without significant decline in exercise performance (although there is some variation on individuals' physical fitness, energy intake during exercise, etc.). Aerobic capacity of children with CF over a long period of time is less researched, indicating the dearth of data regarding longitudinal assessment of peak $\dot{\text{V}}{\text{O}}_{2}$. Klijn et al. investigated the longitudinal relationship between peak $\dot{\text{V}}{\text{O}}_{2}$, pulmonary function, and body composition in 65 children with mild CF lung disease (10.5 ± 2.9 years of age; forced expiratory volume in 1 s (FEV_1_) 92.6 ± 20.5% predicted) over two years. The authors found that longitudinal changes in lung function were associated with functional changes in peak $\dot{\text{V}}{\text{O}}_{2}$ and to a lesser extent with fat free mass (FFM). While FFM is a critical determinant of maximal exercise capacity, FFM may be important for maintaining overall functional capacity and to improve long-term outcome in CF [26, 27]. Peak $\dot{\text{V}}{\text{O}}_{2}$ during maximal exercise is an established prognostic marker in CF [21–23]. In particular, longitudinal data of peak $\dot{\text{V}}{\text{O}}_{2}$ in children with CF indicate a decline in peak $\dot{\text{V}}{\text{O}}_{2}$ over time; however, those with higher peak $\dot{\text{V}}{\text{O}}_{2}$ values have a prolonged survival [23]. In comparison to healthy subjects, children with CF show reduced maximal exercise performance, decreased respiratory function, malnutrition, and physical inactivity as well as intrinsic abnormalities of the skeletal muscle, all likely to be contributing factors [28, 29]. Diminished efficiency of mitochondrial oxidative Adenosine Triphosphate (ATP) synthesis or abnormalities in myofibril mechanics may also affect maximal exercise capacity in CF [26, 30]. It has also been reported that peak $\dot{\text{V}}{\text{O}}_{2}$ and early oxygen consumption ($\dot{\text{V}}{\text{O}}_{2}$) recovery are significantly related in children with chronic lung disease, implying that the greater the aerobic fitness, the faster the rate of recovery following exercise [31]. Hebestreit et al. reported slower $\dot{\text{V}}{\text{O}}_{2}$ kinetics at the onset of exercise in children with CF compared to healthy children, probably due to an impairment in oxygen delivery [32]. 

CF lung disease is often associated with physical inactivity and deconditioning. Improvements in CPF require individual dosages of training stimuli and vary among individuals. However, the optimal training modes for patients with CF have not yet been identified. It has been demonstrated that the time spent on physical activity in children with CF is similar to that of healthy children, but healthy children spend more time exercising at vigorous intensities [33]. In contrast, Selvadurai et al. observed differences in activity behaviours between prepubertal and pubertal children with CF of different stages of disease severity and their healthy peers using accelerometry and activity diaries. Prepubertal and pubertal children with mild CF lung disease were more active compared to their healthy controls. Interestingly, between prepubertal children with moderate-to-severe CF lung disease and a control group no differences were found. Gender differences occurred between pubertal girls and boys with CF, as girls' habitual physical activity levels were significantly lower. However, compared to healthy controls, pubertal children with moderate to severe CF disease were significantly less active [34]. Gender differences in habitual physical activity levels between healthy girls and boys are well known, not only when comparing to chronically diseased children [35, 36].

Several studies investigating the effects of exercise training and rehabilitation programmes in CF showed increased peak $\dot{\text{V}}{\text{O}}_{2}$ and peak minute ventilation (peak ${\dot{\text{V}}}_{\text{E}}$) values, and reduced heart rates (HR) at submaximal workloads, indicating improved CPF [11, 16, 19, 25, 37]. Interestingly, the observed training effects are independent of patients' disease severity. Thus, even patients with severe CF lung disease are able to improve their CPF. Furthermore, it has been established that children with CF had higher activity levels, greater aerobic fitness, enhanced nutritional status, and significantly lower progression of disease [34]. 

Gulmans et al. investigated the effects and acceptability of a 6-month home-based cycling programme in 14 children (age 14.1 years) with mild-to-moderate CF lung disease [38]. The programme consisted of 5 ergometer cycling sessions per week for 20 min each with the training intensity progressively increasing over time. In the first week the training intensity started at 50% of maximal workload, increasing up to 60% in the following weeks until the next measurement was up to 70% of maximal workload. Once a week, the programme was supervised by a physiotherapist. HR was measured every three minutes, and the workload was adjusted to reach target training intensities between 140–160 beats per minute (70%–80% of predicted maximal). After the training period, peak $\dot{\text{V}}{\text{O}}_{2}$ per kg body mass as well as per kg FFM increased significantly. Moreover, leg muscle strength for knee extensors and ankle dorsiflexors significantly increased during the training period. Unfortunately, the acceptability of the programme was low, probably due to the somewhat “monotonous” cycling programme. In general, adherence to treatments is challenging in CF, especially adherence to regular exercise and physical activity. However, the participation in the prescribed exercise training programme has been shown to increase perceived competence and self-esteem in the children as important mediators to adherence behaviour. This seems of particular relevance for patients in terms of maintaining an active lifestyle and adherence to training into the long term. We suggest a training programme that should focus on the individual interests of the children including preferences for sport activities. The study may have benefited by including different activities to maintain motivation and adherence to training [38].

Furthermore, in a 12-week randomised and controlled study following a standardised anaerobic training programme in eleven children with CF, a significant increase in peak $\dot{\text{V}}{\text{O}}_{2}$ (mL·min^−1^) in the training group was observed. The supervised training consisted of two 30–45 min exercise sessions per week of special anaerobic exercises, for example, sprints, chest passes, exchange runs, and so forth. all performed at near maximum intensities. The detailed training programme can be found elsewhere: (http://www.chestjournal.org/content/full/125/4/1299/DC1). In contrast, peak $\dot{\text{V}}{\text{O}}_{2}$ was significantly decreased in the control group (*N* = 9). After a 12-week follow up all parameters concerning aerobic fitness decreased to pretraining levels [16]. 

Schneiderman-Walker et al. investigated the long-term effects of regular exercise training in a 3-year study. Children and adolescents with mild to moderate CF lung disease aged 7–19 years were randomly assigned to either an exercise intervention or control group. The exercise group was instructed to perform a minimum of 20 minutes in their favourite endurance-related activities three times per week. In addition, the patients were advised to monitor their HR while exercising at a training intensity of 70%–80% of maximum HR. Evaluation of training and monitoring was done during telephone and clinical conversations. Exercise capacity was measured during cycle ergometry. At the end of the intervention, no significant differences in the annual rate of decline in peak $\dot{\text{V}}{\text{O}}_{2}$, peak heart rate (peak HR), and peak ${\dot{\text{V}}}_{\text{E}}$ between either group were found. However, the intervention group demonstrated a significantly lower annual decline in FEV_1_ and vital capacity (VC). Patients in the exercise group remained compliant during the study period and reported better well-being [18]. 

#### 2.1.2. Anaerobic Responses to Exercise

Anaerobic activities are characterised by high intensities of short durations (lasting typically up to 30 s), being predominantly fuelled by nonoxidative sources of ATP resynthesis. Previous studies investigating anaerobic performance of children and adults with CF showed lower values compared to healthy subjects [39–41]. Furthermore, children with CF with higher levels of habitual activity have been found to possess increased anaerobic power during the Wingate Anaerobic Test (WAnT). These higher levels of activity are likely influenced by several factors such as the extent of malnutrition, muscle mass, mitochondrial abnormalities, and CF genotype [29, 30, 34, 40, 42, 43]. 

Boas et al. found lower power outputs in the WAnT in 41 male adolescents with CF compared to healthy controls [42]. Subgroup analysis of subjects with CF revealed that nutritional status and sexual maturation rather than pulmonary function may affect anaerobic exercise performance. In their observation, subjects with higher salivary testosterone (>4.0 ng·dL^−1^) had higher power outputs in both absolute terms and relative to lean body mass compared to subjects and lower salivary testosterone values (<4.0 ng·dL^−1^). Furthermore, body mass index (BMI) was associated with higher anaerobic performance, indicating higher power output values. Categorising the patients regarding their pulmonary function showed no differences, however, this population had almost normal lung function values (FEV_1_, 90.2 ± 24.2% predicted). 

Another study showed significant improvements in anaerobic parameters in the WAnT following a 12-week individualised anaerobic training programme. Eleven children with CF lung disease trained 30–45 minutes two times per week. Peak power and mean power per kilogram FFM significantly increased in the training group, as anaerobic parameters did not change in the control group. After a 12-week followup period, anaerobic performance and quality of life remained improved whereas most outcome parameters decreased to pretraining levels [16]. 

In another related study, Selvadurai et al. investigated the relationship between fitness and CF genotype in 79 children with a wide range of disease severity. The class of CF transmembrane conductance regulator (CFTR) mutation was significantly correlated with anaerobic and aerobic performance, BMI, and pulmonary function. Patients with class I and II CFTR mutation had the lowest peak anaerobic power in the WAnT compared to those with classes III, IV, and V CFTR mutations, respectively [43]. 

Unfortunately, most training studies in CF are of short duration, and longitudinal data are lacking, especially in the paediatric population. Longitudinal and controlled studies are required to verify training effects in association with disease progression in CF and decline in pulmonary function and peak $\dot{\text{V}}{\text{O}}_{2}$. Studies, documenting less or no effects after exercise training on CPF, are most likely due to a low number of study subjects, lack of a control group or lack of training supervision, and poor compliance [25, 44]. Further research is warranted to assess the effects of different training methods longitudinally (aerobic versus anaerobic training versus a combination of both training regimes). More detailed information regarding training protocols (intensity, duration, and rest periods) would be advantageous to therapists and health care providers working with children with CF. Furthermore, when working with children the fun component needs to be emphasised, while group activities may be the preferred choice rather than isolated training (i.e., cycling or stepping), infection control permitting. Trampolining, for example, as a popular although controversial activity, has been discussed in the paediatric population and CF lung disease, especially due to the increased risk potential for injuries [45–47]. However, providing there is particular focus on safety issues and supervision, trampolining can be an effective adjunct to regular airway clearance techniques or even as part of physiotherapy in CF [48]. In habitual daily life patients with CF are faced with certain barriers and challenges according to their participation in regular exercise and habitual physical activity [14]. Parents should be encouraged to exercise with their children and serve as role models (i.e., aiming to increase habitual physical activity levels). Based on the training studies available in children and adolescents with CF, there is no definitive evidence regarding the sustainability of training effects in the long term (i.e., months to years).

### 2.2. Possible Risks Associated with Exercise Training in CF

Specific risks of exercise training in CF are presented in the table. During prolonged exercise, as a consequence of excessive sweat and sodium losses, children with CF are advised to drink repeatedly (i.e., every 15–20 min), and ideally including approximately 50 mmol·L^−1^ sodium chloride in their drink [49]. Especially, in hot conditions, children may underestimate their fluid needs with the risk to undergo excessive dehydration [50]. 

Certain sports in this population should also be viewed with caution. For example, CF patients with portal hypertension with significant enlargement of spleen and liver should, as a precaution, be advised against participation in contact sports. Patients participating in physical activities which take place at high altitudes (e.g., skiing) should be monitored, especially if the patient is already hypoxic. Furthermore, episodes of acute right heart failure induced by a combination of altitude and high intensity exercise have been documented [51]. During diving activities air trapping could occur in patients with lung disease, on ascent the air expands, with risk of development of pneumothorax [52]. Exercise-induced hypoxemia is a potential hazard for patients with CF, thus participation in high-intensity exercise needs consideration. However, soon after the termination of high intensity efforts, SaO_2_ returns to pre-exercise levels, while there is no evidence that brief exercise-induced hypoxemia causes damage to the child with CF [53]. Cough with sputum may be observed during exercise in patients; however, this is indicative of airway clearance. 

General guidelines for clinical exercise testing [54, 55] and training in patients with chronic disease are beyond the scope of this paper and can be found elsewhere [49]. The table summarises a selection of recommended exercises, including training intensities for patients with CF. The optimal training mode for CF patients has yet to be defined. Our recommendations (see Table 1) are based on training studies available, as well as, our own clinical and practical experience in the work with children and adolescents with CF. We support the approach of combining endurance and strengthening activities as well as exercises to improve flexibility, balance, and motor skills [7]. In addition to exercise training, an active lifestyle is generally recommended to improve exercise capacity, for example, using stairs instead of an elevator. We therefore recommend including a detailed description of training methods and parameters (duration, intensity, frequency, and rest periods) in future studies. In particular, this may help comparing results of different training studies. Despite the beneficial effects of regular (structured) exercise, habitual physical activity levels (activities that can easily incorporated in daily life) have been shown to contribute to the overall health in children with CF.

## 3. Conclusion

Physical activity is well recognized as part of health care and rehabilitation programmes in CF; however the relationships between activity, fitness, and health are still poorly understood. Higher levels of habitual activity have been associated with higher aerobic and anaerobic capacity, a better quality of life, and improved survival. However, longitudinal studies are needed to support and justify programmes of regular exercise on the overall health status of children and adolescents with CF. To date, there is no evidence that children and adolescents with CF should not be physically active in daily life. Moreover, as part of their CF management, it would be desirable to inform patients, and their parents about the beneficial effects of exercise, but also its potential risks, and additionally provide patients with training recommendations. Ideally, these would be based on previously performed standard clinical exercise testing. It is necessary to instruct clinicians, health care support staff, patients and parents regarding adequate exercises and sports activities during rehabilitation programmes that can be implemented in normal daily life. 

## Figures and Tables

**Table 1:** General exercise and training recommendations.

	Patients with mild to moderate CF lung disease	Patients with severe CF lung disease
Recommended activities	Cycling, walking, hiking, aerobics, running, rowing, tennis, swimming, strength training, climbing, roller-skating, (trampolining)	Ergometric cycling, walking, strengthening exercises, gymnastics, and day-to-day activities

Method	Intermittent and steady-state	Intermittent

Frequency	3–5 times per week	5 times per week

Duration	30–45 minutes	20–30 minutes

Intensity	70%–85% HRmax; 60%–80% peak $\dot{\text{V}}{\text{O}}_{2}$; LT; GET	60%–80% HRmax; 50%–70% peak $\dot{\text{V}}{\text{O}}_{2}$; LT; GET

Oxygen supplementation	Indicated, if SaO_2_ drops below 90% during exercise	Indicated, if SaO_2_ drops below 90% during exercise (cave: resting hypoxia)

Activities to avoid	Bungee-jumping, high diving, and scuba diving	Bungee-jumping, high diving, scuba diving, and hiking in high altitude

Potential risks associated with exercise, and training	Dehydration	
	Hypoxemia	
	Bronchoconstriction	
	Pneumothorax	
	Hypoglycaemia*	
	Hemoptysis	
	Oesophageal bleedings	
	Cardiac arrhythmias	
	Rupture of liver and spleen	
	Spontaneous fractures**	

HRmax: maximum heart rate; peak $\dot{\text{V}}{\text{O}}_{2}$: peak oxygen consumption; LT: lactate threshold; GET: gas exchange threshold; SaO_2_: oxygen saturation.

*Depending on the existence of an impaired glucose tolerance.

**Depending on the existence of untreated CF-related bone disease.

## Abbreviations

CF: Cystic fibrosis
CPF: Cardiopulmonary fitness
Peak $\dot{\text{V}}{\text{O}}_{2}$: Peak oxygen consumption
FEV_1_: Forced expiratory volume in 1 s
FFM: Fat free mass
ATP: Adenosine Triphosphate
$\dot{\text{V}}{\text{O}}_{2}$: Oxygen consumption
peak ${\dot{\text{V}}}_{\text{E}}$: Peak ventilation
HR: Heart rate
peak HR: Peak heart rate
VC: Vital capacity
WAnT: Wingate Anaerobic Test
BMI: Body mass index
CFTR: Cystic fibrosis transmembrane conductance regulator
SaO_2_: Oxygen saturation
${\dot{\text{V}}}_{\text{E}}$: Expired ventilation
$\dot{\text{V}}\text{C}{\text{O}}_{2}$: Volume of carbon dioxide
ECG: Electrocardiogram
CO_2_: Carbon dioxide
FVC: Forced vital capacity
GET: Gas exchange threshold
MVV: Maximum voluntary ventilation
f_R_: Respiratory rate
P_ET_CO_2_: End-tidal pressure of carbon dioxide.
